# Immunotherapy with DNA vaccine and live attenuated rubella/SIV gag vectors plus early ART can prevent SIVmac251 viral rebound in acutely infected rhesus macaques

**DOI:** 10.1371/journal.pone.0228163

**Published:** 2020-03-04

**Authors:** Konstantin Virnik, Margherita Rosati, Alexei Medvedev, Aaron Scanlan, Gabrielle Walsh, Frances Dayton, Kate E. Broderick, Mark Lewis, Yvonne Bryson, Jeffrey D. Lifson, Ruth M. Ruprecht, Barbara K. Felber, Ira Berkower

**Affiliations:** 1 Laboratory of Immunoregulation, Division of Viral Products, Office of Vaccines, Center for Biologics, Food and Drug Administration, Silver Spring, Maryland, United States of America; 2 Human Retrovirus Section, Vaccine Branch, Center for Cancer Research, National Cancer Institute at Frederick, Frederick, Maryland, United States of America; 3 Inovio Pharmaceuticals, Inc., Plymouth Meeting, Pennsylvania, United States of America; 4 BioQual, Inc., Rockville, Maryland, United States of America; 5 Department of Pediatrics, Division of Infectious Disease, David Geffen School of Medicine at UCLA, Los Angeles, California, United States of America; 6 AIDS and Cancer Virus Program, Frederick National Laboratory for Cancer Research, Frederick, Maryland, United States of America; 7 University of Louisiana at Lafayette, New Iberia Research Center, New Iberia, Louisiana, United States of America; 8 Human Retrovirus Pathogenesis Section, Vaccine Branch, Center for Cancer Research, National Cancer Institute at Frederick, Frederick, Maryland, United States of America; Emory University, UNITED STATES

## Abstract

Anti-retroviral therapy (ART) has been highly successful in controlling HIV replication, reducing viral burden, and preventing both progression to AIDS and viral transmission. Yet, ART alone cannot cure the infection. Even after years of successful therapy, ART withdrawal leads inevitably to viral rebound within a few weeks or months. Our hypothesis: effective therapy must control both the replicating virus pool and the reactivatable latent viral reservoir. To do this, we have combined ART and immunotherapy to attack both viral pools simultaneously. The vaccine regimen consisted of DNA vaccine expressing SIV Gag, followed by a boost with live attenuated rubella/gag vectors. The vectors grow well in rhesus macaques, and they are potent immunogens when used in a prime and boost strategy. We infected rhesus macaques by high dose mucosal challenge with virulent SIVmac251 and waited three days to allow viral dissemination and establishment of a reactivatable viral reservoir before starting ART. While on ART, the control group received control DNA and empty rubella vaccine, while the immunotherapy group received DNA/gag prime, followed by boosts with rubella vectors expressing SIV gag over 27 weeks. Both groups had a vaccine “take” to rubella, and the vaccine group developed antibodies and T cells specific for Gag. Five weeks after the last immunization, we stopped ART and monitored virus rebound. All four control animals eventually had a viral rebound, and two were euthanized for AIDS. One control macaque did not rebound until 2 years after ART release. In contrast, there was only one viral rebound in the vaccine group. Three out of four vaccinees had no viral rebound, even after CD8 depletion, and they remain in drug-free viral remission more than 2.5 years later. The strategy of early ART combined with immunotherapy can produce a sustained SIV remission in macaques and may be relevant for immunotherapy of HIV in humans.

## Introduction

Anti-retroviral therapy (ART) drug cocktails have controlled HIV replication, lowered viral load to undetectable levels, prevented disease progression, and reduced HIV transmission [1]. However, it is not currently possible to cure HIV infection with ART drugs alone. In adults, even after years of complete viral control by ART, withdrawal of ART leads to viral rebound within four to six weeks in most cases [2, 3]. The drugs must be taken for life. In a rare instance early ART has delayed viral rebound for up to 27 months following ART withdrawal, as occurred following perinatal transmission to the “Mississippi baby” [4, 5], suggesting the possibility of long-term viral control.

The retroviral replication cycle includes obligatory integration into host cell DNA. Most infected cells are productively infected, but in some cells, the integrated provirus can remain latent for years [6]. The provirus can be replicated along with the host cell DNA, in which it is integrated, without expressing viral proteins [7–9], remaining biologically silent and immunologically invisible. These latently infected cells form a viral reservoir that persists indefinitely despite successful ART [6]. When ART is withdrawn, latent proviruses can be activated, and this reseeds the infection all over again. In the case of the “Mississippi baby”, the long delay between ART release and viral rebound suggests that early ART may control replicating virus, limit the size of the reservoir, and preserve immune function. This scenario would potentially allow immunotherapy to work, by providing pre-emptive immune surveillance of reactivating viruses from the latent reservoir that persists during ART and long after treatment is discontinued. We have tested this hypothesis by combining early ART treatment with immunotherapy in the SIV-infected rhesus macaque model [10].

In our study, we exposed macaques to high dose mucosal challenge with pathogenic SIVmac251. We gave the infection a three-day head start before initiating ART, using a triple drug regimen employed previously by Whitney et al. [11, 12]. Under these conditions three to seven days were sufficient to establish a rebound competent viral reservoir in most animals, as shown by rebound viremia when ART was withdrawn after 6 months of treatment. The effect of immunotherapy on the rebound competent viral reservoir can be measured as a delay or reduction in the frequency of viral rebounds in the vaccine group as compared to controls.

Immunotherapy consisted of a DNA vaccine expressing SIV gag [13, 14] followed by a boost with live attenuated rubella vectors expressing SIV gag [15, 16]. The rubella vectors are based on the licensed vaccine strain RA27/3 [17], which has demonstrated its safety and potency in millions of children as part of the MMR vaccine. It is even recommended for those HIV-positive children who can respond to it [18]. The vectors stably expressed SIV Gag antigens as small as a few T cell epitopes and as large as SIV p27^Gag^ protein [16]. The vectors grow well in rhesus macaques, and they are potent immunogens when used in tandem with DNA vaccines [19]. Together, they elicit high titers of Gag-specific antibodies that persist for over one year, as well as Gag-specific T cells [20]. The longevity of the vaccine induced Gag-specific T cells suggests their potential utility for long term immunosurveillance.

During the study, viral replication, as measured by plasma viremia, was well controlled while on ART, but ART withdrawal led to prompt viral rebound in three out of four control animals, with two animals progressing to AIDS. In contrast, in the immunotherapy group only one animal had a viral rebound after ART withdrawal. The other three vaccinated animals were protected against viral rebound, and they remain virus-free after more than 2.5 years without ART. Additional studies are planned to confirm these results and to determine the optimal timing and targets of vaccine-induced protection.

The results in the macaque model of SIV infection could also apply to human infection with HIV. Neonatal infection can be diagnosed and treated with ART, starting as early as 30 hours of life [4] Subsequently, immunotherapy could control the latent reservoir. The therapeutic window for rubella-based immunotherapy could be after 6 to 9 months, when maternal antibodies have waned, and before 5 to 9 years, when most non-immunized children become naturally infected with rubella. Rubella vaccine is suitable for use in children, including those with HIV infection [18, 21]. If early ART plus immunotherapy can control the latent SIV reservoir in macaques, the same strategy may allow humans to control the HIV reservoir, prevent viral rebound, and achieve sustained remission, independent of drugs.

## Results

### Study design

**Fig 1:**
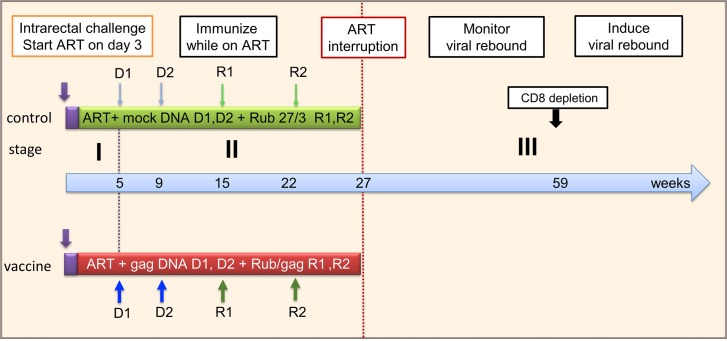
Study plan. Stage I: high-dose mucosal challenge of rhesus macaques with SIVmac251 by the intra-rectal route. ART was delayed until day 3 to allow establishment of the latent viral reservoir. Stage II: the vaccine group was immunized with DNA/gag expression plasmid followed by rubella/gag vectors, while on ART. Controls received control DNA and empty rubella vaccine. Stage III: after 27 weeks, ART was withdrawn, and the animals were followed for viral rebound. Subsequently, CD8^+^ T cells were depleted to release latent virus.

We tested the combined effect of early ART plus immunotherapy on recent SIV infection using an experimental design with three stages (Fig 1). In Stage I, the animals were exposed to a high dose intrarectal mucosal challenge with SIVmac251. SIVmac251 is a fully pathogenic virus swarm [22, 23] that is sensitive to T cell immunity but relatively insensitive to Env-specific neutralizing antibodies [24]. The high- dose intrarectal challenge was 50 times the titered dose that transmitted infection to about half of the animals following a single rectal challenge.

No drugs were given for the first three days of infection, in order to permit viral dissemination and establishment of a post-ART rebound competent viral reservoir. After three days, we started daily ART with a triple drug cocktail and continued this for 26 weeks. While under ART treatment, the virus was well controlled, and the animals remained aviremic (< 50 SIV RNA copies/mL).

During Stage II, the animals were immunized while virus was suppressed on ART. The control group (n = 4) received control DNA and conventional rubella vaccine with no insert. The immunotherapy group (n = 4) were primed with two doses of plasmid DNA vaccine coding for SIV Gag, given by electroporation at weeks 5 and 9 following infection. This was followed by two doses of live rubella vectors expressing SIV gag, at weeks 15 and 22 post infection. The rubella/gag vectors were described previously [15, 16]. One rubella vector expressed four predominant Gag T cell epitopes in tandem and was called BC-sGag2. The other vector expressed the entire SIV p27^Gag^ plus p2^Gag^ protein.

After the last immunization, we continued ART treatment for another 5 weeks. This was intended to allow the immune response to reach peak levels before ART interruption. The entire infection, ART treatment, and immunization phase spanned 27 weeks.

During Stage III, we withdrew ART and monitored plasma viremia for signs of a viral rebound. If a three day head start was sufficient to seed the viral reservoir, this would be shown by viral rebound in the control group within 4 to 8 weeks [11, 12]. If immunotherapy had an antiviral effect, it could prevent or delay viral rebound in the vaccine group. If any animals did not rebound after 32 weeks without ART, we would provoke rebound by depletion of CD8^+^ T cells.

### Rubella gene expression is insensitive to ART

Rubella vector growth and protein expression were tested for sensitivity to the triple ART drug regimen in cell culture (tenofovir, emtricitabine, and dolutegravir). It seemed unlikely that these drugs would interfere with rubella growth as they target the retroviral reverse transcriptase and integrase, which are not found in rubella. But it was important to know this, since the potency of rubella vectors appears to depend on vector replication and the level of antigen expression [19]. Rubella vector expression was measured by western blot of rubella structural proteins C and E1. As a positive control, we measured the effect of ART on HIV replication, using HIV pseudovirions expressing luciferase. As shown in S1 Fig, rubella vector growth and protein expression were insensitive to the triple ART cocktail even at 125 times the dose needed to reduce HIV replication by 99%. Similar results were obtained for Tenofovir alone as monotherapy.

### Immune response to the rubella vector and gag insert

**Fig 2:**
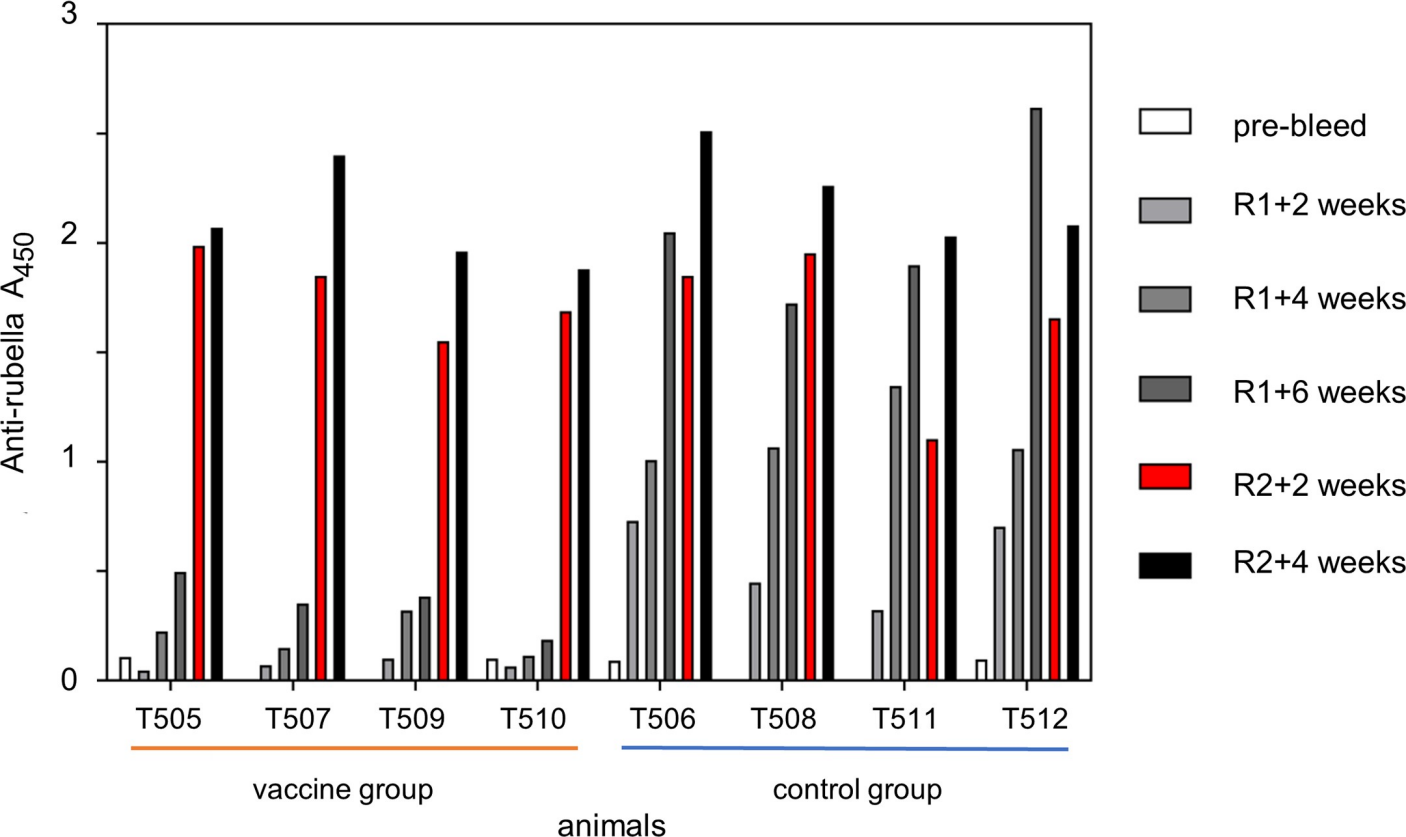
Immune response to rubella antigens. The anti-rubella response indicates a vaccine “take” in all macaques, as detected by ELISA. The control group (right) received empty rubella vaccine and responded to the first dose of rubella (R1 + 6 weeks). The vaccine group (left) responded to the second dose of rubella/gag vectors (R2 + 2 and 4 weeks in red).

Both groups were immunized with rubella vectors, with or without a gag insert. We measured antibodies to the rubella vector as a sign of a vaccine “take” (Fig 2). The control group responded to the first dose of conventional rubella vaccine and showed a typical stepwise increase in anti-rubella antibody titers from weeks 2 to 6 after a single immunization. In contrast, the vaccine group showed weaker rubella specific responses to the first dose of rubella/gag vectors and needed a second dose to produce a strong and rising response between weeks 2 and 4 after the boost. The need for a boost and resulting strong response to it is commonly found with rubella vectors expressing exogenous vaccine antigens, which tend to grow slower [19]. The strong anti-rubella response in both groups indicated a vaccine “take” while the animals were receiving ART.

**Fig 3:**
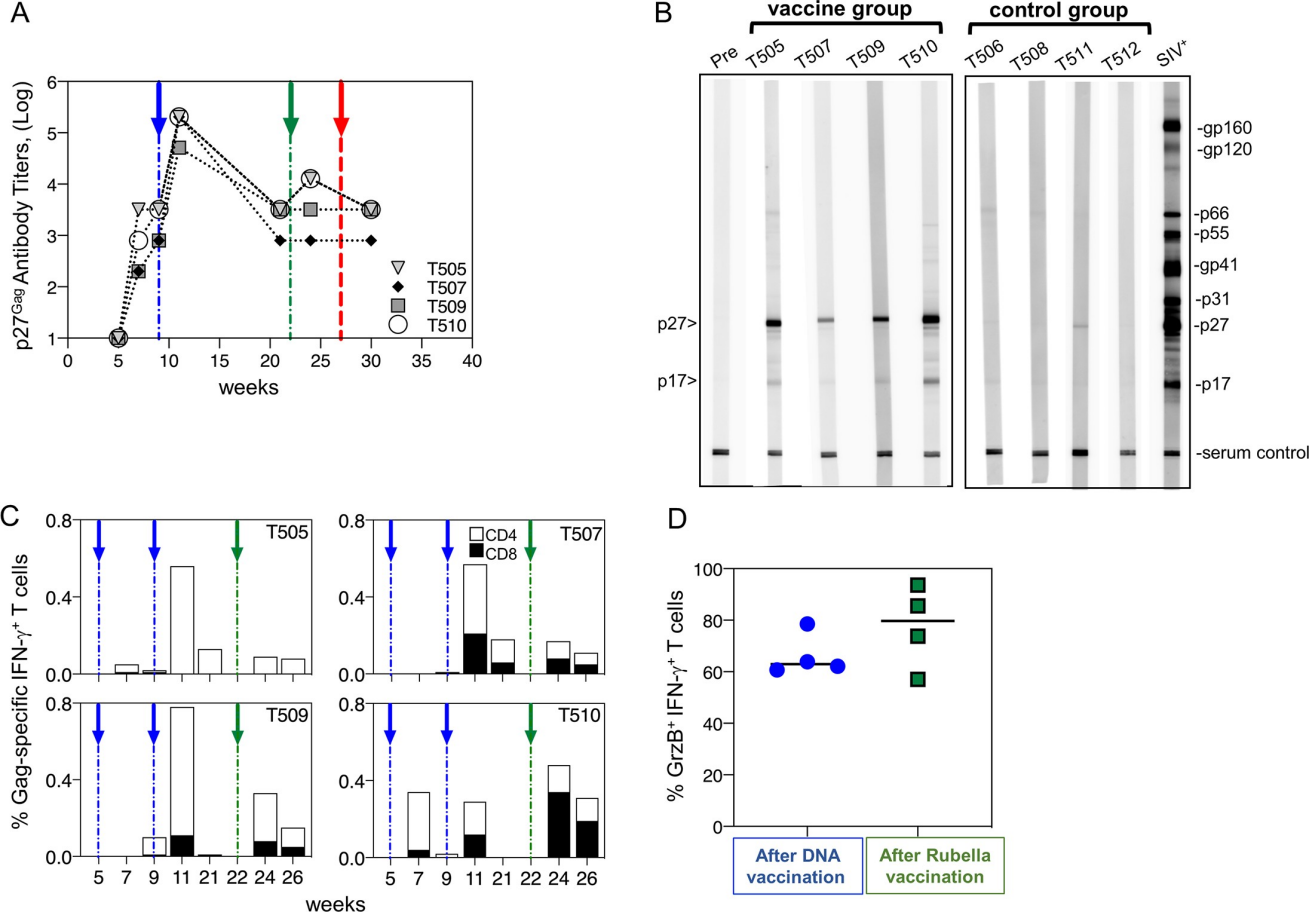
Immunogenicity of the rubella/gag vaccine insert. (A) ELISA measurements of Gag antibody titers upon vaccination of macaques with *gag* DNA prime and Rubella/*gag boost*. (B) Antibodies to SIV Gag were detected by Western blot on Zeptometrix antigen strips one week after stopping ART. At this time (week 28), all animals were PCR negative. The vaccine group produced anti-p27^Gag^ antibodies, and some had anti-p17^Gag^, while the controls were all negative. (C) Measurements of Gag CD4^+^ and CD8^+^ Gag T cell responses upon vaccination. (D) Cytotoxic T cells (GrzB^+^), as percentage of Gag-specific IFN-γ+-producing cells, were measured after DNA vaccination (week 11) and after rubella/gag boost in the 4 animals of the vaccine group. The blue arrows indicate DNA vaccination; green arrows indicate rubella vector vaccination; red arrows indicate time of ART withdrawal.

We tested for the presence of antibodies to Gag and Gag specific T cells as a sign of vaccine immunogenicity (Fig 3). ELISA analysis confirmed the development of anti-Gag antibodies (Fig 3A) as a result of DNA and rubella vaccinations. ZeptoMetrix western blot analysis was used to measure antibody specificity at one week after ART interruption (Fig 3B). All animals were still aviremic (< 50 SIV copies/mL) at this time (six weeks after the last immunization). The western blot indicates the immune status of each macaque in the weeks leading up to viral rebound. All four animals in the vaccine group showed antibodies to p27^Gag^, and three had antibodies to p17^Gag^ as well (animals T505, T509, and T510) as a result of the vaccinations. None of the control animals showed antibodies to SIV Gag (animals T506 to T512). A faint p27^Gag^ band in control T511 may be the first sign of viral rebound in this macaque (confirmed two weeks later by RT PCR) (see below). No antibodies to Env were detected at this early time point.

Analysis of the T cell responses showed induction of robust Gag-specific T cells in PBMC upon vaccination with DNA and rubella/gag vectors. The responses were heterogeneous, mediated primarily by CD8^+^ T cells in T507 and T510 and primarily by CD4^+^ T cells in T505 and T509 (Fig 3C). All animals showed predominantly Gag-specific T cells with effector memory (CD3^+^ CD95^+^ CD28^-^) phenotype (S2 Fig). Rubella gag vectors strongly boosted the Gag specific T cell responses in T509 and T510. Vaccinations with gag DNA induced high levels of cytotoxic (GrzB+, IFN-γ+) T cell responses which were maintained upon rubella vector boost (Fig 3D). Thus, the vaccine regimen induced a high frequency of Granzyme B+/IFN-γ+ Gag-specific T cells.

### Viral suppression and rebound

**Fig 4:**
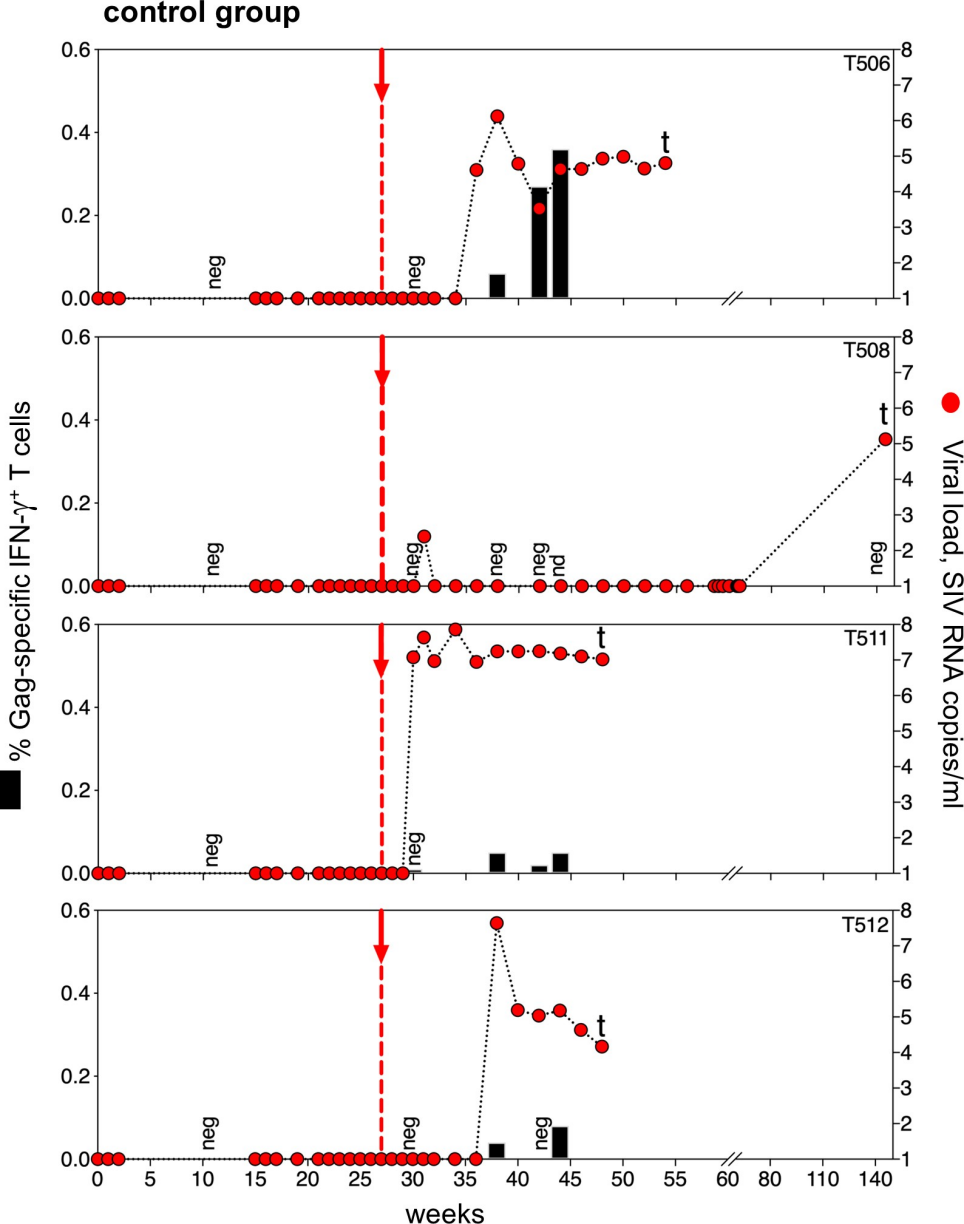
Viral rebound and T cell responses in the control group. Each plot shows an individual animal. Red arrows indicate ART withdrawal, and red circles show the viral rebounds. T cell responses to Gag are shown as black bars. Macaques T506, T511, and T512 showed viral rebound in the expected time range, while macaque T508 had a prolonged delay before viral rebound, suggesting a correspondingly small viral reservoir. Macaques (T508, T511 and T512) were euthanized due to an AIDS-like illness (indicated by t).

**Fig 5:**
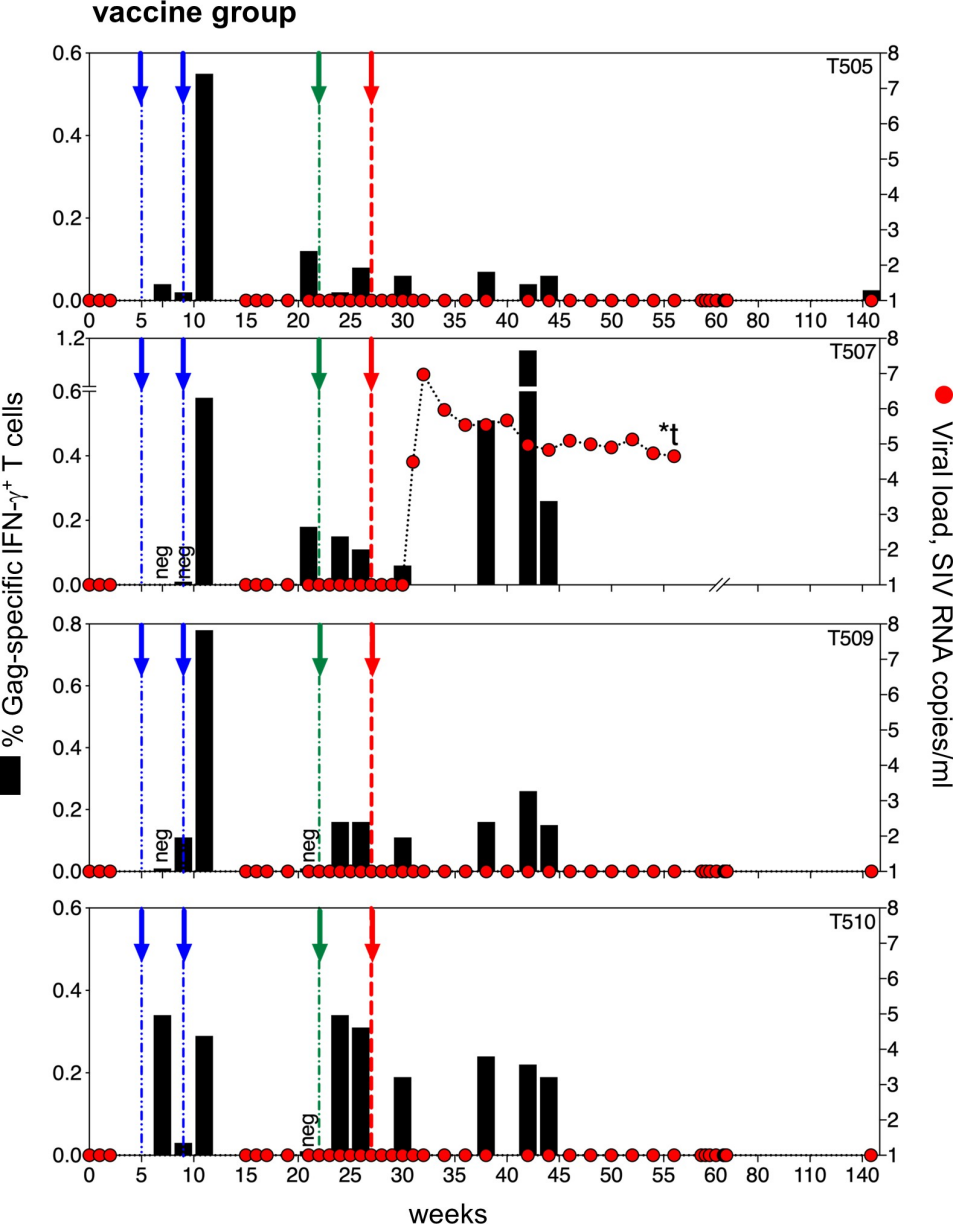
Viral rebound and T cell response in the vaccine group. Arrows indicate gag DNA vaccine given by electroporation (blue), last dose of rubella/gag vectors (green), and ART withdrawal (red). Viral loads (red circles) show viral rebound in just one out of four macaques (T507). The other three vaccinated macaques have remained virus-free for over 2 years after ART withdrawal. T cell responses to Gag are shown in black bars. Two animals with the most robust and durable T cell responses (T509 and T510) had complete control of viremia. Macaque T507 was euthanized before developing AIDS (indicated by t). Macaque T505 was euthanized for reasons unrelated to AIDS (indicated by *t).

After ART interruption, we monitored rebound infection by measuring viral load, Gag-specific T cell response (Figs 4 and 5), Env-specific T cell response (S3 Fig) and CD4^+^ T cell count (S4 Fig). In the control group, all four macaques showed viral suppression to < 50 copies/mL plasma for 27 weeks while on ART (Fig 4). Following ART withdrawal, all control animals showed viral rebound after 3, 9, 11 or 120 weeks, with 2 animals (T511 and T508) progressing rapidly to AIDS, with pulmonary signs and CD4^+^ T cell depletion within a few weeks of the onset of post-ART viremia (S4A Fig).

Control animals T506 and T512 showed a similar pattern of SIV control on ART, followed by viral rebound after ART withdrawal (Fig 4). In T506, plasma viremia remained below 50 SIV RNA copies/ml until 9 weeks after stopping ART (red arrow) and then rose quickly to 10^6^ copies/ml. Two weeks later, T506 showed a robust T cell response, characterized by CD8^+^ T cells with effector memory phenotype (S2A Fig), and the viral load declined by about one log. In T512, virus rebounded at 11 weeks with a peak viral load of 10^8^ SIV RNA copies/ml that declined to 10^5^ copies/ml when low levels of Gag-specific T cells appeared.

Control animal T511 showed a different pattern of viral rebound, starting 3 weeks after ART interruption. Plasma viremia reached 10^7^ SIV RNA copies/ml, yet the T cell response was minimal, and the macaque failed to control viremia at all. This animal behaved like a subset of macaques with progressive SIV infection that seem to be unable to mount a robust T cell response to the challenge virus. This animal progressed rapidly to AIDS and was euthanized. In the three control macaques with early viral rebound, circulating CD4^+^ T cells were markedly depleted within a few weeks of the onset of viremia (S4A Fig).

Control animal T508 had a viral blip in plasma RNA (250 copies/ml) at 4 weeks off ART, but plasma viremia was < 50 copies/mL in all subsequent tests. In addition, lymph nodes and PBMC were analyzed for SIV DNA and cell-associated RNA at different time points throughout the study (S5 Fig) using a highly sensitive quantitative RT-PCR assay (threshold 2 copies/10^6^ cell equivalents). All samples were negative, except a single lymph node (LN) sample collected at week 19 post ART release which was weakly positive for cell-associated viral RNA (six copies per 10^6^ cells), (S1 Table). Remarkably, viral rebound occurred between week 83 and week 120 following ART withdrawal, with viremia of 1.3 X 10^5^/ml and progression to AIDS by week 120. The prolonged delay of viral rebound in this animal suggests seeding of a small viral reservoir. But the rapid progression to AIDS shows the fragility of viral control. This may reflect the nearly absent immune response to viral antigens when the virus is fully suppressed on ART.

The immunotherapy group was monitored over time for rebounding viral load and T cell responses to Gag antigens (Fig 5). The virus was well controlled in all animals while on ART. Following ART withdrawal, three out of four vaccinated macaques (T505, T509 and T510) continued to control the virus for over 2 years without ART. Animals T509 and T510 (Fig 5) had plasma viremia < 50 copies/mL while on ART and no measurable viral rebound for 2.5 years after ART withdrawal. Both animals made a T cell response to the DNA/gag vaccine that declined before the rubella/gag vectors were given. They responded to the rubella/gag vector boost (green arrows) and maintained T cell immunity for at least 20 weeks after immunization, including the first 15 weeks after ART withdrawal. Similarly, macaque T505 (Fig 5) controlled virus while on ART. Despite a modest T cell response to vaccine, it also controlled viral load for two years after ART withdrawal.

In contrast, one vaccinated animal T507 (Fig 5) failed to control the virus after ART withdrawal. Rebound viremia occurred four weeks after ART release, and the course of viremia was indistinguishable from that of control animals. T507 had robust predominantly CD8^+^ Gag T cell responses upon DNA vaccination but showed a weak response to rubella/gag vectors. Its rebound coincided with the nadir of T cell immunity. The viral load reached 10^7^ SIV RNA copies/mL and then declined to 10^5^ copies/mL. Despite a limited initial response to rubella vectors, this animal was capable of mounting a strong anamnestic CD8^+^ T cell response to Gag during the viral rebound, when Gag specific T cells reached 1.2% of total circulating T cells. Interestingly, the viral rebound did not affect CD4^+^ T cell count of T507, which was similar to those of the aviremic vaccine group animals (S4B Fig). Taken together, the data support the concept that Gag-specific T cells are important for controlling viremia and preventing viral rebound.

We compared the T cell responses of the vaccine group with viral outcome to understand the immunological basis of protection. While there are too few examples to reach a firm conclusion, it appears that the low point of T cell immunity and the area under the curve of T cell response may predict the outcome of infection. The only rebound in the vaccine group occurred in one of the two vaccinated macaques (T505 and T507) with lower T cell responses at the time of ART withdrawal, when the nadir of T cell immunity for macaque T507 at week 30 coincided with the start of his viral rebound. Conversely, the two vaccinated animals (T509 and T510) with the most robust and durable T cell responses at the time of ART withdrawal were the ones protected from viral rebound (Fig 5).

### CD8^+^ cell depletion and the mechanism of protection

We studied the three vaccinated animals and one control animal that did not show viral rebound by week 32 after ART withdrawal to understand the mechanism of viral suppression. Prior studies have shown that CD8^+^ T cells play a key role in controlling the virus after infection and delaying disease progression [25]. We used an approach that could provoke a viral rebound in the aviremic animals. CD8^+^ T cells and NK cells were depleted with monoclonal anti-CD8 antibody cM-T87R1 at week 59, and we monitored potential viral rebound. Under these conditions, viral rebound would indicate that suppression is a dynamic state that depends on continued immune surveillance by T cells. Absence of viral rebound would suggest complete viral remission after prolonged ART plus immunotherapy.

Complete depletion of circulating CD8^+^ T cells for 2 to 4 weeks after monoclonal antibody treatment is shown in S6 Fig. There was no evidence of viral rebound in any of the 4 animals during or after CD8 depletion. However, it was not possible to determine if this treatment also depleted lymph node CD8^+^ T cells, especially GALT T cells. The absence of viral rebound in blood was documented using a highly sensitive RT-PCR assay (threshold 2 SIV RNA copies/mL plasma) (S1 Table). We further measured cell-associated viral RNA and DNA from lymph nodes and from PBMC collected at 1 week and 2 weeks post CD8 depletion. We were unable to detect rebounding virus in any of these samples (S1 Table).

The lack of latent virus reactivation suggests possible eradication of viable provirus in the combined ART plus immunotherapy group. In that case, after 6 months of ART plus immunotherapy, CD8^+^ Tcells may not be needed for transient control of viral rebound, although continuous CD8 surveillance would be needed for sustained control. Another possible explanation for the delayed viral rebound is that a pathogenic virus like SIVmac251 may favor cell death over viral latency, resulting in a smaller latent reservoir.

We also monitored viral load in lymph nodes during early infection (4 weeks) and early ART, when circulating virus was undetectable and prior to the start of immunotherapy. We used the sensitive droplet digital PCR (ddPCR) method [26] to measure viral load (S2 Table) while on ART. The positive controls were PBMC taken from the same animals after viral rebound, and these were uniformly positive by ddPCR. The test samples included five animals that would later rebound after ART withdrawal and three animals that did not rebound. All 8 samples were negative by ddPCR, indicating that viral rebound can occur, starting from a latent viral reservoir that is below the limit of detection of 4 copies per 10^6^ cell equivalents (S2 Table). We repeated the assay with PBMC taken after 7 weeks on ART, when the animals had received the first dose of DNA vaccine or a sham DNA vaccine. These PBMC samples gave results similar to those for the lymph nodes collected earlier.

## Discussion

We studied early ART plus immunotherapy with DNA vaccine and rubella vectors as a strategy for producing viral remission in the SIV challenge model in rhesus macaques. Previously, ART alone was known to reduce the plasma viral load to undetectable levels, yet it could not cure infection. HIV and SIV can persist as at least two subsets, the actively replicating pool and the latent viral reservoir. We hypothesized that viral remission would require treatment of both subsets. ART would control replicating virus, while immunotherapy could elicit durable immune surveillance of the latent viral reservoir. Immunotherapy consisted of a DNA gag vaccine followed by live attenuated rubella vectors expressing SIV Gag.

We infected eight macaques with SIVmac251 and initiated ART 3 days later, to allow sufficient time to seed the viral reservoir. If we started ART too early, it could reduce the number of viral rebounds in the control group after ART release [10, 12]. But if we waited too long, the expanded viral reservoir could overwhelm immune control in the vaccine group. The animals received ART daily for 27 weeks. Half of the animals were immunized with SIV gag while on ART, and half of the animals received control vaccine. After 27 weeks, ART was discontinued, and viral rebound was monitored. The control group showed two patterns of viral rebound, with 3 animals rebounding within 3–12 weeks and one exceptional animal (T508) that rebounded after 2.3 years and progressed to AIDS. The vaccine group showed durable protection against viral rebound in three out of four macaques.

The major findings of this paper suggest that rubella/gag vectors may be favorable for use in immunotherapy of children infected at birth such as the “Mississippi baby”, and perhaps others like her. The key to successful treatment may be starting ART early, when viral load is low and the immune response is intact. Babies born to HIV seropositive mothers could be diagnosed early and started on ART. The “Mississippi baby” was diagnosed and treated within 30 hours of life [4, 5]. She maintained good virological control for 18 months while on ART. Surprisingly, she continued to control the virus for another 27 months after ART interruption. The long delay in viral rebound suggests that early ART had limited the latent viral reservoir size and activity but ultimately, control of viremia was lost. Similarly, a control macaque (T508) was treated early after infection (day 3) and controlled viremia long after ART withdrawal. After 18 months of ART interruption, SIV rebounded. Like the “Mississippi baby”, macaque T508 had a very small latent reservoir. Importantly, this observation shows that prolonged time after ART interruption is critical to the assessment of the potency of an immunotherapeutic intervention.

Rubella/gag immunotherapy can follow infection by at least 15 to 22 weeks and still prevent viral rebound. Early ART could control the replicating virus, limit the size of the latent reservoir, and preserve immune function. Immunotherapy by 6 to 9 months of age could activate immune surveillance of the latent reservoir and prevent viral rebound. By this time (6 to 9 months) pre-existing maternal antibodies to rubella will generally wane in the newborn. This should provide a window of opportunity for immunotherapy with rubella/gag vectors, lasting until MMR vaccine is given at age 12 to 15 months (in developed countries) or natural rubella infection at age 5 to 9 in countries that do not have a rubella immunization program.

While rare in North America, about 12,000 children are born with HIV infection each month in Africa [27]. DNA/gag vaccine prime followed by rubella/HIV gag vector boost should be safe for this group. The vectors are derived from the live attenuated rubella vaccine strain RA27/3 that is given to millions of healthy children each year as part of the MMR vaccine. Rubella vaccine is even recommended for HIV infected children, if they are well enough to respond to it [18].

Viral rebound in the infected macaque model is a sensitive measure of the size and activity of the latent viral reservoir [6, 10, 11]. In our study, all four animals in the control group tested negative for SIV while on ART, based on sensitive qPCR and ddPCR of lymph nodes. Yet, following ART withdrawal, all four macaque controls rebounded to high viral loads. This agrees with previous studies showing that viral rebound can be seeded from a latent viral reservoir that was below the limit of detection by sensitive analytical methods. Viral rebound may be the most sensitive measure of the size and viability of the latent viral reservoir.

A great deal has been learned about viral reservoirs by studying the SIV rebound model in macaques. Tsai et al originally demonstrated that ART can be delayed up to one day after infection and still prevent seeding of the latent reservoir for SIVmne (SIV isolated from a pig-tailed macaque) in cynomolgus macaques [10]. Whitney et al confirmed this for SIVmac in rhesus macaques and showed that three days’ delay before starting ART would allow establishment of the viral reservoir [11, 12]. Even after 6 months of complete virological control on ART, with no circulating virus detected, four of four [11] or five of five macaques rebounded within a few weeks of ART interruption [12]. In general, rebound competency depends on several factors, including: dose of SIV, route of infection, timing of ART initiation, and duration of ART treatment, as well as the challenge virus.

In theory, we can distinguish different levels of viral suppression, based on the response to CD8^+^ T cell depletion. At the lowest level of immune control, the rate of removal of actively infected cells would equal the rate of viral propagation. In this case, CD8^+^ depletion could quickly tip the balance toward viral rebound. At the next level, the CD8^+^ T cells would eliminate actively infected cells as fast as they can replicate, but it would not control latent infections. Depletion of CD8^+^ T cells would remove immune control and permit random viral blips, leading to viral rebound over time. At the highest level of control, the CD8^+^ T cells would eradicate live virus and prevent reactivation of provirus, leading to complete viral remission.

In our study, we tested the level and type of viral suppression by depleting CD8^+^ T cells in the four animals that were still in remission after 31 weeks without ART (three vaccinees and one control). Treatment with monoclonal anti-CD8 antibody depleted all circulating CD8^+^ cells for over one month. However, CD8 depletion did not lead to virus rebound in any animal. In the protected macaques, early ART plus immunotherapy appeared to reduce the viral reservoir below a critical size, leading to complete viral remission. However, our inability to detect the low levels of the viral reservoir before and after immunotherapy prevents a clear demonstration of the extent of depletion of the viral reservoir in each animal due to immunotherapy. If complete remission of SIV infection can be achieved, it will depend on the duration of infection before ART is initiated, virulence of the challenge strain, and strength of the immune response to vaccine.

These findings are consistent with prior findings with CMV-SIV gag vectors. The immunized macaques showed SIV viral blips at early timepoints, but these became infrequent over time [28]. The instability of proviral sequences as well as the low fraction of full-length proviral DNA was also shown for SIV by Pion et al. [29]. These authors inoculated purified, supercoiled SIVmac239 plasmid DNA intramuscularly into rhesus macaques, which resulted in high levels of viremia. After only a few weeks, the single molecular species was replaced by genomic diversity due to multiple deletions in the proviral DNA.

In contrast to our results, reversible viral suppression by CD8^+^ cells has been reported by two labs [30, 31]. These were both SHIV challenge studies, where immunotherapy consisted of neutralizing monoclonal antibodies with or without innate immune modulators. Nishimura et al showed control of viremia when two neutralizing monoclonal antibodies were given at the time of SHIV challenge [30]. This protected state was reversed by CD8^+^ T cell depletion, but then restored when CD8^+^ T cells recovered. Borducchi and Barouch showed similar results when animals were treated with ART followed by monoclonal anti-Env antibody and a TLR7 agonist [31].

A number of live viral vectors have been studied for HIV or SIV prevention and cure. Two promising vectors include live rhesus CMV expressing multiple SIV antigens [28], and live attenuated VSV expressing SIV or HIV gag [32, 33]. The CMV vectors cause chronic infection and produce long-lasting protection against SIV. This correlates with unconventional MHC restricted CD8^+^ T cell responses induced by the vaccines [28, 34]. They have consistently protected about 55% of macaques when given prior to SIV infection [28].

Sneller et al. [3] conducted a clinical trial of immunotherapy with a DNA vaccine prime followed by a VSV/gag vector boost in HIV-infected adults. The subjects started ART during the acute or early phase of HIV infection, within 28 days (median) after diagnosis. They were well controlled on ART for two to four years before entering the study. Immunotherapy consisted of DNA vaccine given by electroporation, alternating with two doses of live attenuated VSV/gag vectors. Despite treatment, viral rebounds in the vaccine group were no different from the controls when ART was withdrawn. This study was quite different from ours, including the longer delay in starting ART (28 days in humans vs. 3 days in macaques), duration of ART (2 to 4 years vs. 6 months), and different viral vectors used for immunization (VSV/gag vs. rubella/gag). Functional cure of HIV in adults may be very difficult to achieve unless we can reduce the delay between infection, diagnosis and treatment.

Our results with SIVmac251 infection in macaques may guide the search for a permanent remission of human infection with HIV. Perhaps the most important result is to know that early ART and T cell immunotherapy can work together to produce durable remission of infection under optimal conditions. We will continue to use the macaque model to extend the timing, breadth and duration of the immune response that is needed for viral remission following acute infection.

## Materials and methods

### Animals

The study used juvenile rhesus macaques of Indian origin at 3 years of age and weighing between 3.2 and 4.3 Kg. All animals were MHC typed, and animals positive for Mamu-A*01, B*08, and B*17 MHC type were excluded from the study to avoid protective effects of MHC on disease progression.

The study was done at BioQual, Inc, in a research facility that is fully accredited by the Association for Assessment and Accreditation of Laboratory Animal Care International (AAALAC). All experimental procedures were approved by the Institutional Animal Care and Use Committee and were done in compliance with the Guide for the Care and Use of Laboratory Animals.

Non-human primates were housed in mobile modular stainless-steel caging with minimum floor space of 6.0sq ft and ability to link together for enrichment and social housing purposes. Animal cages were washed down on a daily basis and sanitized at least every two weeks along with scheduled room sanitization. Temperature and humidity were kept at a constant level in accordance with guide recommendations and were documented twice daily on room activities logs. Non-human primates were fed Purina Lab Diet old-world primate chow twice daily and are supplemented once daily with additional fruit or vegetable. Additional enrichment treats were provided on a minimum weekly basis and typically much more frequently for positive reinforcement. Enrichment was overseen by an on-site behaviorist and was driven by the BIOQUAL Environmental Enrichment Plan (BEEP). Primary goals of this plan are to provide social housing when possible, in-cage enrichment items (toys), various foraging tasks & food enrichment. Caging was also equipped with perches for each animal. Animal care staff monitored the health and well-being of the animals on a daily basis. The criteria used to make such assessments included evaluation for mentation, appetite, stool, nasal discharge, blood in cage, pain/distress, and overall appearance. Rounds were performed twice daily by trained husbandry or technical personnel. Any findings were reported back to the attending veterinarian for evaluation and treatment.

All animals passed a screening complete physical exam and a veterinary evaluation of their medical history including serology. Animals were negative for antibodies to rubella, herpes B (one exception), STLV-1, SIV, and SRV (type D, 1, 2, and 3 by serology/PCR). The animals were negative for Shigella, Salmonella, Yersinia, Campylobacter and tuberculosis and free of intestinal pathogenic parasites.

All animals received ketamine anesthesia (10 mg/kg/im) for restraint during immunization, bleeding and taking oral swabs.

The 8 macaques were divided into two groups of four each. Both groups were infected intrarectally with SIVmac251, and both started ART on day 3 of infection. The vaccine group received DNA prime and rubella/gag boost. The control group received control DNA and conventional rubella vaccine RA27/3 with no insert.

### SIV Infection

The SIVmac251 challenge strain [23] was obtained from Dr. Nancy Miller at NIAID. The challenge stock, called “Desrosiers 2010 Day 8”, was derived by infection of rhesus PBMCs. Animals were infected by high dose rectal challenge with 1 ml of a 1:18 dilution of this highly pathogenic challenge stock. This was approximately 50 times the dose needed to infect half of the macaques in low dose challenge studies. It has reliably infected nearly all macaques in previous high-dose challenge studies.

### ART treatment

Combination ART therapy was the same as reported previously [11]. It consisted of tenofovir (20 mg/kg), emtricitabine (30 mg/kg) and dolutegravir (2.5 mg/kg). Triple drug therapy was delayed until day 3 post infection to allow the viral reservoir to become established [11, 12].

### Immunizations

The DNA vaccine consisted of 2 mg DNA coding for SIV gag p57 (plasmid 206S) and MCP3p39 (plasmid 209S) [14] plus 0.2 mg of DNA encoding for macaque IL-12 (plasmid AG157) [35]. The control group received empty plasmid DNA (pCMV-Kan) without an insert plus 0.2 mg of rmIL-12 DNA. The DNA vaccine was given twice, at weeks 5 and 9 post infection, by in vivo electroporation with the Elgin 1000 device (Inovio Pharmaceuticals, Inc., Blue Bell, PA), while on ART therapy.

The rubella/gag vaccine consisted of two vectors based on the live attenuated rubella vaccine strain RA27/3, with gag inserts at the structural insertion site. One vector expressed SIV gag p27+p2 [16]. The other vector, called BC-sGag2, expressed four major gag T cell epitopes in tandem [15], including GY9, TE15, CM9 and ME11. All constructs were verified by sequencing. The rubella/gag vectors previously demonstrated avid growth and stable antigen expression in cell culture, as well as growth and immunogenicity in rhesus macaques [19, 20].

The rubella/gag vectors were given twice, at 15 and 22 weeks post infection, while on ART. This was followed by a five-week ART extension, to allow time to reach peak levels of immune surveillance of the active and latent viral reservoirs. The control group received conventional “empty” rubella vaccine RA27/3 with no insert. The dose of each vector was 100,000 PFU, which is about 20 times the recommended dose for humans [18].

### Detection of antibody and T cell responses and viral load

ELISA immunoassays for detecting antibodies to rubella antigens, as a measure of a vaccine “take”, were described previously [19]. The antibody response to the SIV Gag insert was measured by ZeptoMetrix western blot (ZeptoMetrix, Buffalo, NY) using nitrocellulose strips containing SIV_mac_ viral proteins from detergent-disrupted virus particles [36]. Serum from an SIV-infected macaque was the positive control. Gag-specific T cells were assayed by intracellular cytokine staining after antigen stimulation with peptide pools, as described previously [37].

Viral load was routinely determined by RT-PCR of monkey plasma by BioQual, Inc, with a sensitivity of 50 RNA copies/ml. In selected samples, we used a sensitive ddPCR method to detect proviral DNA in PBMC or lymph nodes (S2 Table) [26]. Plasma SIV gag RNA and cell associated SIV gag DNA and RNA in LN and PBMC (S1 Table) were measured using quantitative PCR and RT PCR methods, essentially as described using high sensitivity assay formats [38, 39].

Gag-specific CD4^+^ T cells and CD8^+^ T cells in PBMC were detected by flow cytometry (for further details, refer to Rosati et al. [20]). CD8^+^ T cell depletion was performed by giving DAKO monoclonal antibody at 32 weeks after ART interruption. Following treatment, CD8^+^ T cells were undetectable in all animals for at least the next 2 to 4 weeks.

### Determination that ART has no effect on rubella growth and protein expression

ART effect on rubella replication and protein expression was determined by measuring expression of rubella C and E1 structural proteins in Western blot of infected whole cell lysates treated with various concentrations of antiretroviral drugs. The Western blot procedure has been previously described [16]. Digital images were quantified using GeneSys (v.1.6.1.0) and GeneTools (v.4.3.8.0) software (Syngene/Synoptics Ltd., England). Expression at different drug levels was normalized to the maximum expression, which was set as 100%. The 1X drug concentrations in the combination were 0.4 μM tenofovir, 0.4 μM emtricitabine and 2 nM dolutegravir. Drug levels used in the experiment were multiples of these concentrations, as indicated.

As a positive control, ART inhibition of HIV was measured in the HIV pseudovirion luciferase assay [40] at the same drug concentrations. The pseudovirions displayed X4 tropic HIV (IIIB) envelope glycoproteins and coded for a luciferase reporter gene. The assay was done on U87-CD4^+^ cells expressing CXCR4 coreceptors. Virus infectivity was determined by following the luciferase signal in the absence or presence of drugs. The measured signals were normalized to the maximum signal set as 100%.

## Supporting information

S1 Fig ART does not interfere with rubella growth and protein expression. Rubella replication was measured by expression of C and E1 structural proteins on western blot at various concentrations of ART drugs. Protein expression was normalized to the maximum expression level set as 100%. As a positive control, ART effect on HIV IIIB pseudovirions was measured by luciferase assay. The 1X drug concentrations were 0.4 μM tenofovir, 0.4 μM emtricitabine and 2 nM dolutegravir. Drugs were used at multiples of these concentrations, as indicated. For comparison, pharmacokinetics studies show that peak drug concentrations for tenofovir can reach 4 μM [41]. This corresponds to concentration between 5x and 25x in the figure. ART levels that inhibited HIV by 99% had almost no effect on rubella replication and protein expression.
(PDF)

S2 Fig SIV Gag-specific T cell subsets at various timepoints throughout the study. (A) The control group was monitored at baseline, at week 11 and after ART withdrawal. (B) In the vaccine group, T cell subsets were monitored before and after ART withdrawal (red arrow), after DNA vaccine (blue arrow), and after rubella vectors (green arrow). Left panel: CD4^+^ (open bars) and CD8^+^ (black bars). Right panel: CM CD95^+^ CD28^+^ (light grey bars) and EM CD95^+^ CD28^-^ (grey bars). The grey shaded area is from Fig 3C; neg, negative; nd, not done.
(PDF)

S3 Fig SIV Env-specific T cell subsets after ART interruption. Env specific T cells would indicate a response to rebounding virus, since there was no Env in the vaccine. In both control group and vaccine group T cell subsets were monitored at 3, 11, 15 and 16 weeks after ART withdrawal. Left panel: CD4^+^ (open bars) and CD8^+^ (black bars). Right panel: CM CD95^+^ CD28^+^ (light grey bars) and EM CD95^+^ CD28^-^ (grey bars). The red arrows indicate time of ART withdrawal; neg, negative; nd, not done. The x-axes show weeks of the study.
(PDF)

S4 Fig CD4^+^ T cell measurements throughout the study. CD4^+^ T cells in the control (A) and vaccine (B) groups measured during ART and upon ART withdrawal (red arrows). While on ART, both groups showed preservation of CD4^+^ T cells. After ART withdrawal, (red arrows), these cells declined the most in control monkeys with high viral loads (T506, T511, T512, and eventually T508).
(PDF)

S5 Fig The timeline indicating when high sensitivity PCR and droplet digital PCR assays were performed throughout the study to detect SIV CA-RNA and DNA in LN and PBMC. (PDF)

S6 Fig CD8 depletion and virus load. The plots show the effect of CD8^+^ T cell depletion in the 4 treated animals that did not rebound by week 59 of the study. Virus load did not rebound, despite complete depletion of CD8^+^ T cells. Virus loads were measured using the high sensitivity assay (threshold 2 copies/ml), and levels below threshold were plotted as one copy/ml.
(PDF)

S1 Table SIV DNA and cell-associated (CA)-RNA measured as copies per 10^6^ cell equivalents. (PDF)

S2 Table Viral load in lymph nodes post infection, as measured by ddPCR. (PDF)
