# Supplementary material for: Immunotherapy with DNA vaccine and live attenuated rubella/SIV gag vectors plus early ART can prevent SIVmac251 viral rebound in acutely infected rhesus macaques
Source: PLoS One. 2020 Mar 4;15(3):e0228163. doi: 10.1371/journal.pone.0228163 (PMC7055890; doi:10.1371/journal.pone.0228163)
Supplement: S1 Fig — Rubella replication was measured by expression of C and E1 structural proteins on western blot at various concentrations of ART drugs. Protein expression was normalized to the maximum expression level set as 100%. As a positive control, ART effect on HIV IIIB pseudovirions was measured by luciferase assay. The 1X drug concentrations were 0.4 μM tenofovir, 0.4 μM emtricitabine and 2 nM dolutegravir. Drugs were used at multiples of these concentrations, as indicated. For comparison, pharmacokinetics studies show that peak drug concentrations for tenofovir can reach 4 μM [41]. This corresponds to concentration between 5x and 25x in the figure. ART levels that inhibited HIV by 99% had almost no effect on rubella replication and protein expression. (PDF) [file pone.0228163.s001.pdf]

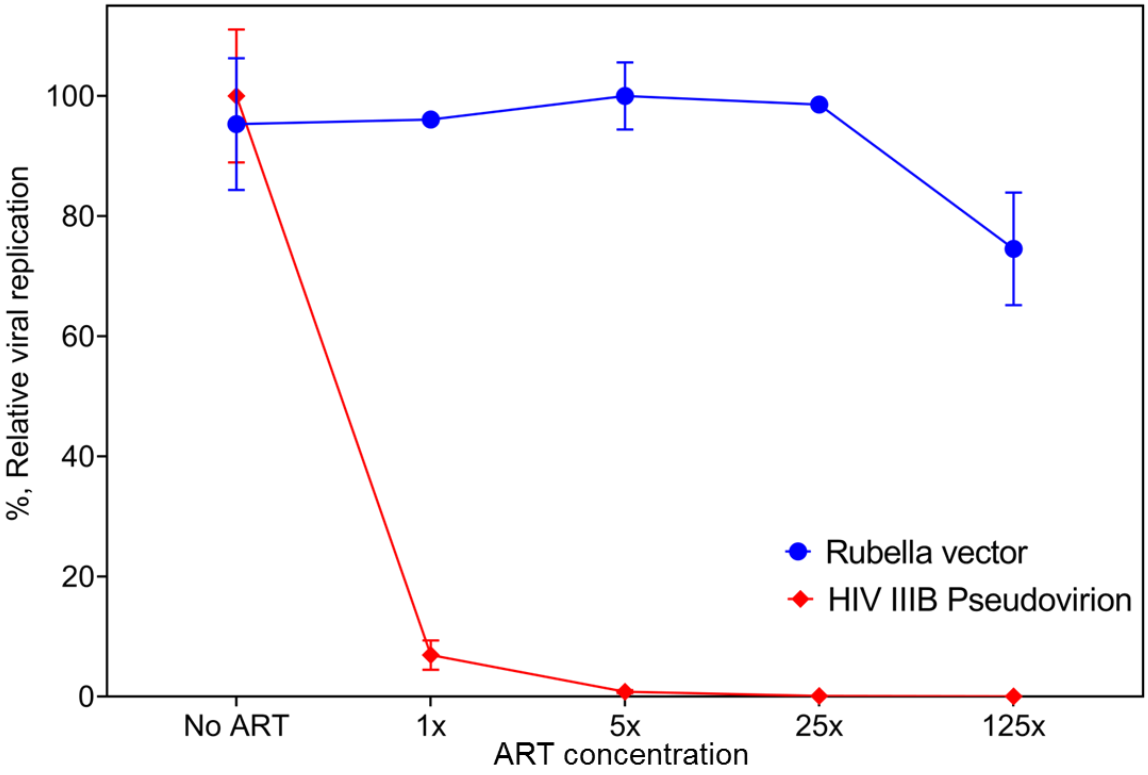

S1 Fig. ART does not interfere with rubella growth and protein expression. Rubella replication was measured by expression of C and E1 structural proteins on western blot at various concentrations of ART drugs. Protein expression was normalized to the maximum expression level set as 100%. As a positive control, ART effect on HIV IIIB pseudovirions was measured by luciferase assay. The 1X drug concentrations were 0.4 µM tenofovir, 0.4 µM emtricitabine and 2 nM dolutegravir. Drugs were used at multiples of these concentrations, as indicated. For comparison, pharmacokinetics studies show that peak drug concentrations for tenofovir can reach 4 µM [41]. This corresponds to concentration between 5x and 25x in the figure. ART levels that inhibited HIV by 99% had almost no effect on rubella replication and protein expression.
