# Supplementary material for: Immunotherapy with DNA vaccine and live attenuated rubella/SIV gag vectors plus early ART can prevent SIVmac251 viral rebound in acutely infected rhesus macaques
Source: PLoS One. 2020 Mar 4;15(3):e0228163. doi: 10.1371/journal.pone.0228163 (PMC7055890; doi:10.1371/journal.pone.0228163)
Supplement: S2 Fig — (A) The control group was monitored at baseline, at week 11 and after ART withdrawal. (B) In the vaccine group, T cell subsets were monitored before and after ART withdrawal (red arrow), after DNA vaccine (blue arrow), and after rubella vectors (green arrow). Left panel: CD4+ (open bars) and CD8+ (black bars). Right panel: CM CD95+ CD28+ (light grey bars) and EM CD95+ CD28- (grey bars). The grey shaded area is from Fig 3C; neg, negative; nd, not done. (PDF) [file pone.0228163.s002.pdf]

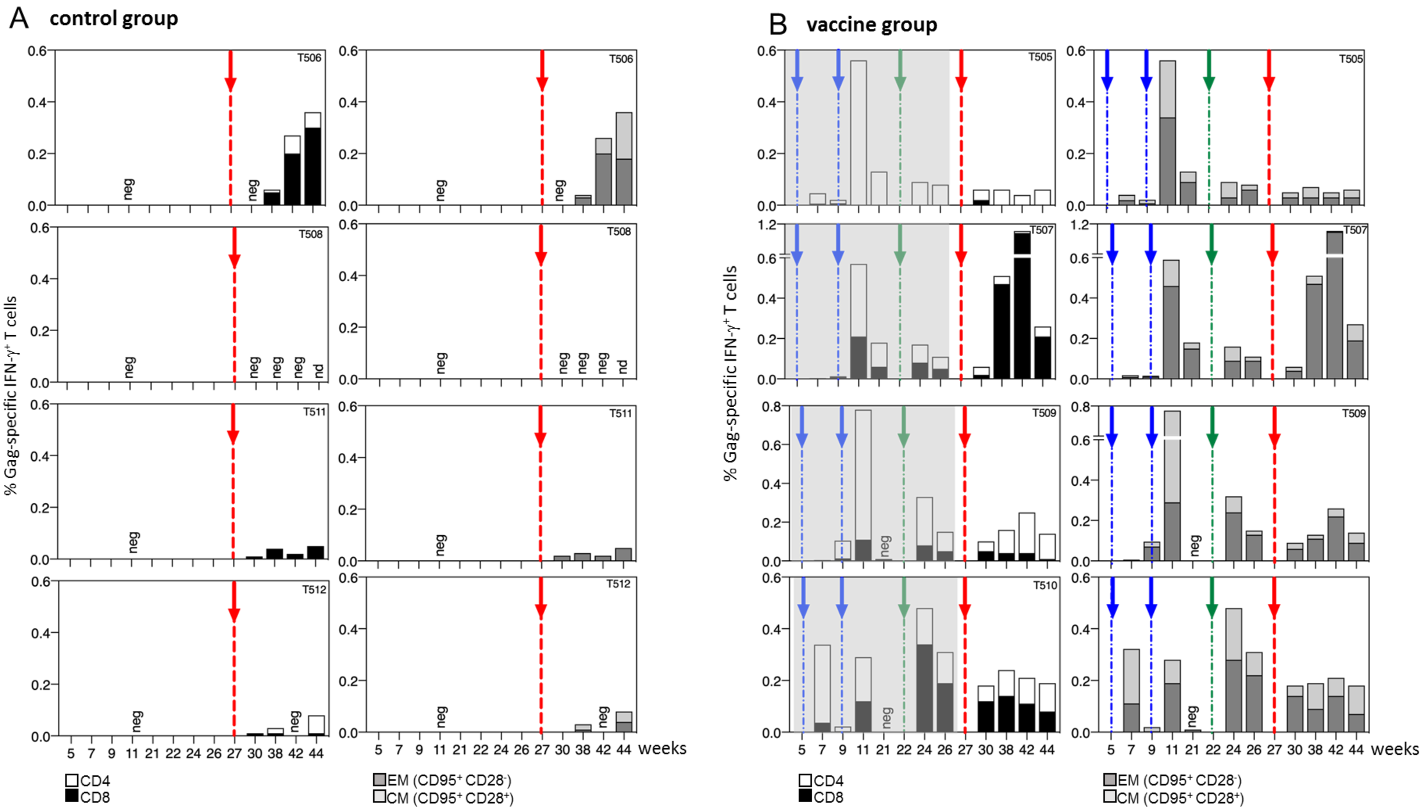

S2 Fig. SIV Gag-specific T cell subsets at various timepoints throughout the study. (A) The control group was monitored at baseline, at week 11 and after ART withdrawal. (B) In the vaccine group, T cell subsets were monitored before and after ART withdrawal (red arrow), after DNA vaccine (blue arrow), and after rubella vectors (green arrow). Left panel: CD4+ (open bars) and CD8+ (black bars). Right panel: CM CD95+ CD28+ (light grey bars) and EM CD95+ CD28- (grey bars). The grey shaded area is from Fig. 3C; neg, negative; nd, not done.
