# Supplementary material for: Immunotherapy with DNA vaccine and live attenuated rubella/SIV gag vectors plus early ART can prevent SIVmac251 viral rebound in acutely infected rhesus macaques
Source: PLoS One. 2020 Mar 4;15(3):e0228163. doi: 10.1371/journal.pone.0228163 (PMC7055890; doi:10.1371/journal.pone.0228163)
Supplement: S3 Fig — Env specific T cells would indicate a response to rebounding virus, since there was no Env in the vaccine. In both control group and vaccine group T cell subsets were monitored at 3, 11, 15 and 16 weeks after ART withdrawal. Left panel: CD4+ (open bars) and CD8+ (black bars). Right panel: CM CD95+ CD28+ (light grey bars) and EM CD95+ CD28- (grey bars). The red arrows indicate time of ART withdrawal; neg, negative; nd, not done. The x-axes show weeks of the study. (PDF) [file pone.0228163.s003.pdf]

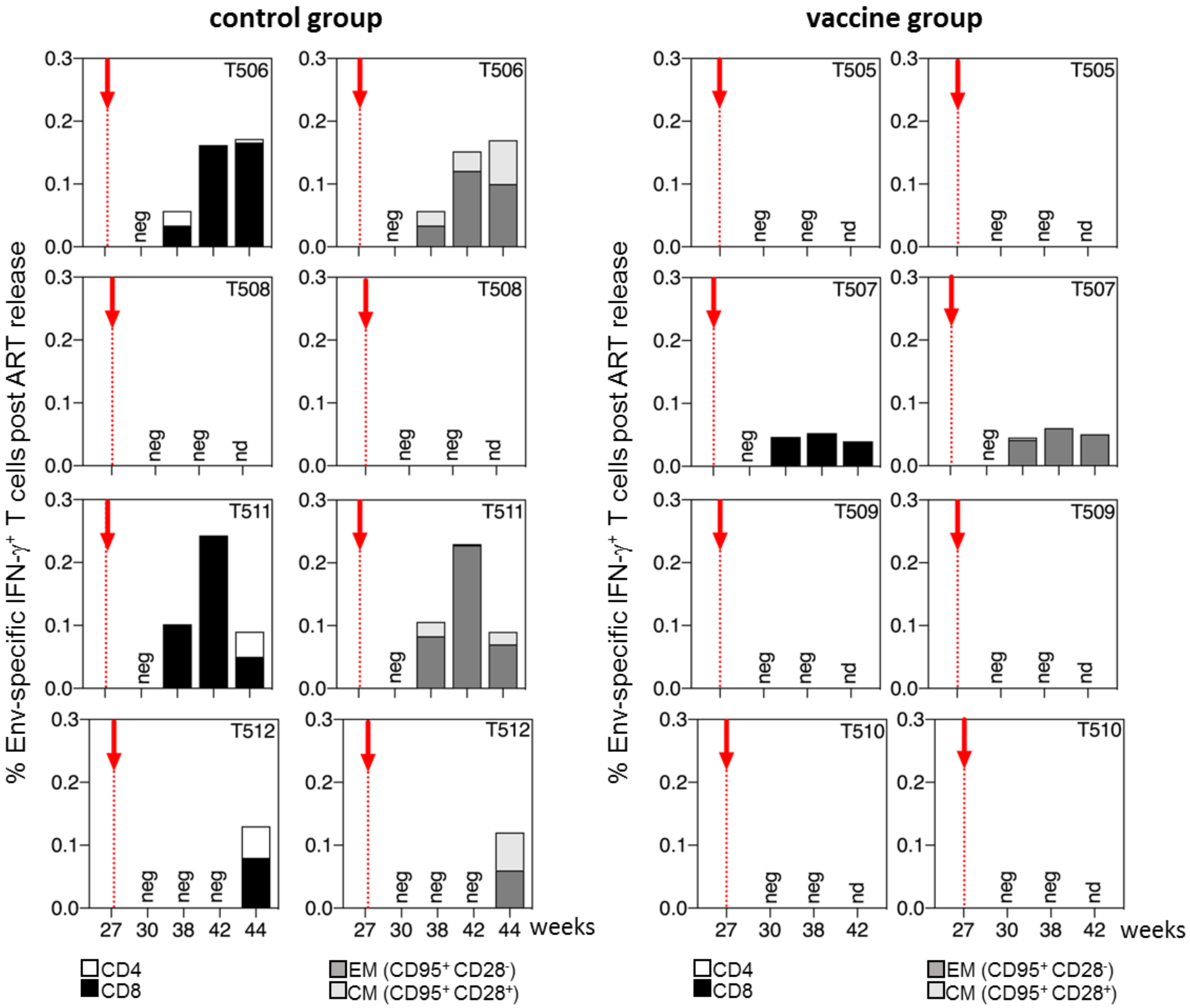

S3 Fig. SIV Env-specific T cell subsets after ART interruption. Env specific T cells would indicate a response to rebounding virus, since there was no Env in the vaccine. In both control group and vaccine group T cell subsets were monitored at 3, 11, 15 and 16 weeks after ART withdrawal. Left panel: $CD4^+$ (open bars) and $CD8^+$ (black bars). Right panel: CM $CD95^+$ $CD28^+$ (light grey bars) and EM $CD95^+$ $CD28^-$ (grey bars). The red arrows indicate time of ART withdrawal; neg, negative; nd, not done. The x-axes show weeks of the study.
