# Supplementary material for: Immunotherapy with DNA vaccine and live attenuated rubella/SIV gag vectors plus early ART can prevent SIVmac251 viral rebound in acutely infected rhesus macaques
Source: PLoS One. 2020 Mar 4;15(3):e0228163. doi: 10.1371/journal.pone.0228163 (PMC7055890; doi:10.1371/journal.pone.0228163)
Supplement: S4 Fig — CD4+ T cells in the control (A) and vaccine (B) groups measured during ART and upon ART withdrawal (red arrows). While on ART, both groups showed preservation of CD4+ T cells. After ART withdrawal, (red arrows), these cells declined the most in control monkeys with high viral loads (T506, T511, T512, and eventually T508). (PDF) [file pone.0228163.s004.pdf]

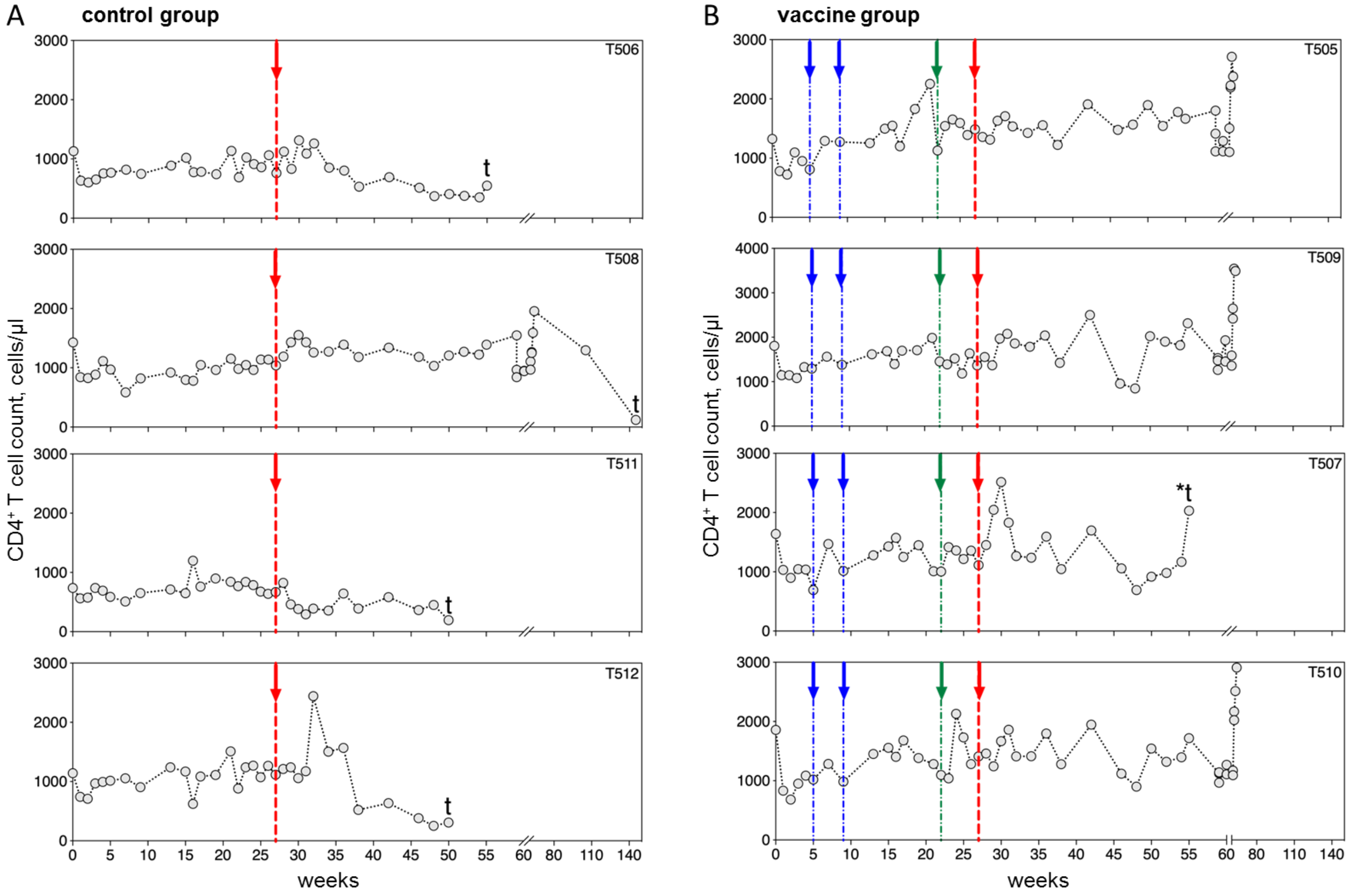

S4 Fig. CD4+ T cell measurements throughout the study. CD4+ T cells in the control (A) and vaccine (B) groups measured during ART and upon ART withdrawal (red arrows). While on ART, both groups showed preservation of CD4+ T cells. After ART withdrawal, (red arrows), these cells declined the most in control monkeys with high viral loads (T506, T511, T512, and eventually T508).
