# Supplementary material for: Immunotherapy with DNA vaccine and live attenuated rubella/SIV gag vectors plus early ART can prevent SIVmac251 viral rebound in acutely infected rhesus macaques
Source: PLoS One. 2020 Mar 4;15(3):e0228163. doi: 10.1371/journal.pone.0228163 (PMC7055890; doi:10.1371/journal.pone.0228163)
Supplement: S5 Fig — (PDF) [file pone.0228163.s005.pdf]

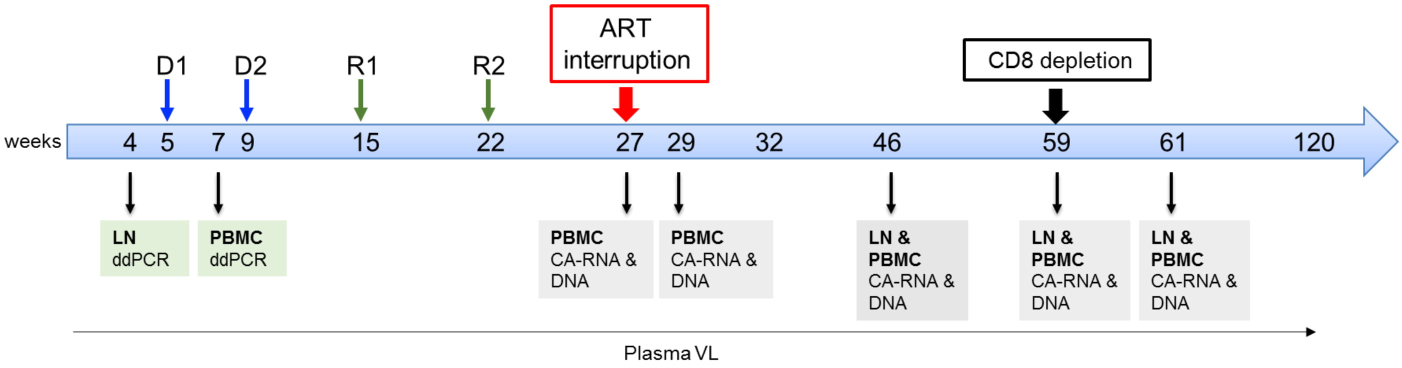

S5 Fig. The timeline indicating when high sensitivity PCR and droplet digital PCR assays were performed throughout the study to detect SIV CA-RNA and DNA in LN and PBMC.
