# Supplementary material for: Immunotherapy with DNA vaccine and live attenuated rubella/SIV gag vectors plus early ART can prevent SIVmac251 viral rebound in acutely infected rhesus macaques
Source: PLoS One. 2020 Mar 4;15(3):e0228163. doi: 10.1371/journal.pone.0228163 (PMC7055890; doi:10.1371/journal.pone.0228163)
Supplement: S6 Fig — The plots show the effect of CD8+ T cell depletion in the 4 treated animals that did not rebound by week 59 of the study. Virus load did not rebound, despite complete depletion of CD8+ T cells. Virus loads were measured using the high sensitivity assay (threshold 2 copies/ml), and levels below threshold were plotted as one copy/ml. (PDF) [file pone.0228163.s006.pdf]

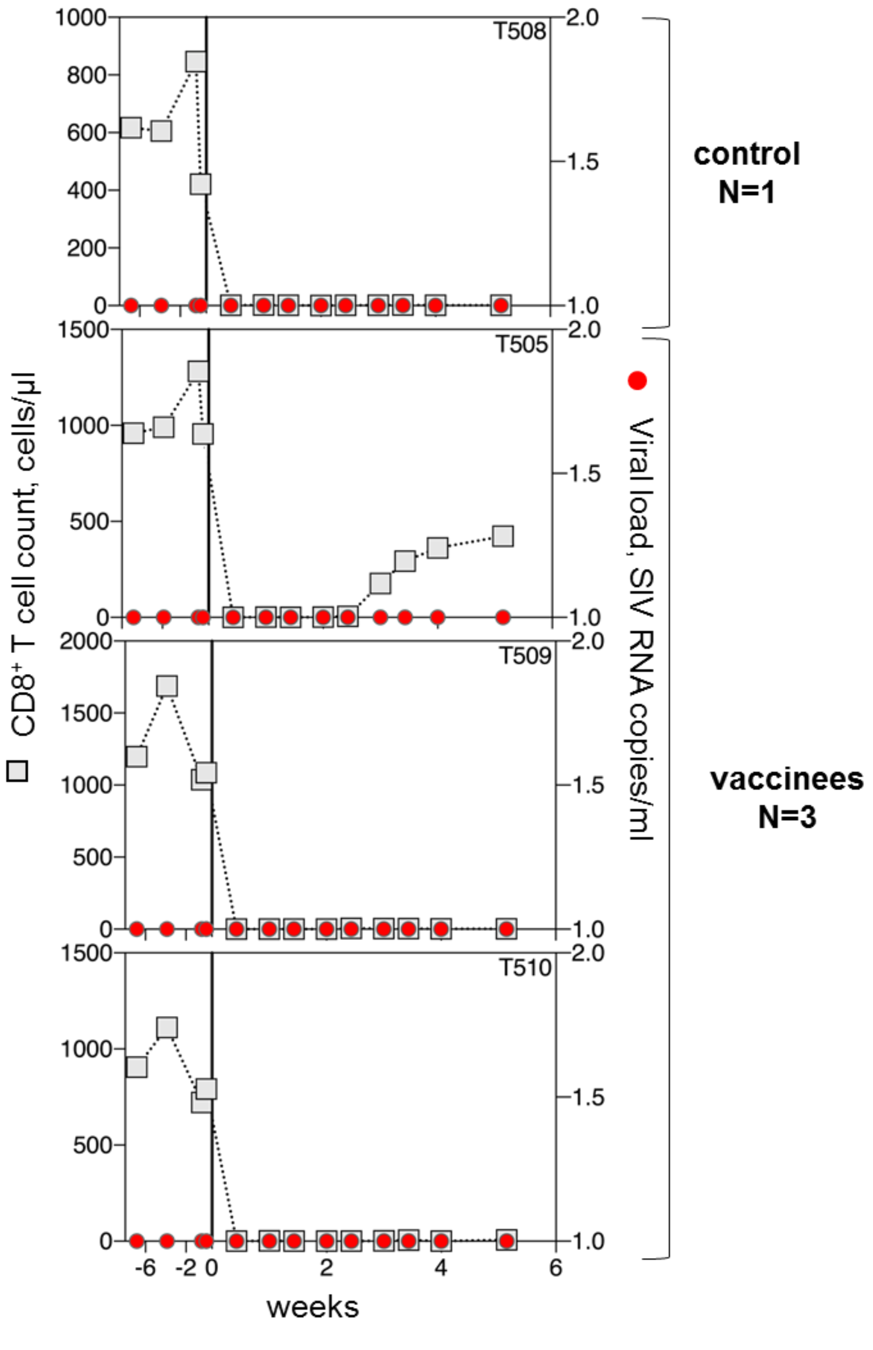

S6 Fig. CD8 depletion and virus load. The plots show the effect of CD8+ T cell depletion in the 4 treated animals that did not rebound by week 59 of the study. Virus load did not rebound, despite complete depletion of CD8+ T cells. Virus loads were measured using the high sensitivity assay (threshold 2 copies/ml), and levels below threshold were plotted as one copy/ml.
