# Supplementary material for: Immunotherapy with DNA vaccine and live attenuated rubella/SIV gag vectors plus early ART can prevent SIVmac251 viral rebound in acutely infected rhesus macaques
Source: PLoS One. 2020 Mar 4;15(3):e0228163. doi: 10.1371/journal.pone.0228163 (PMC7055890; doi:10.1371/journal.pone.0228163)
Supplement: S1 Table — (PDF) [file pone.0228163.s007.pdf]

S1 Table. SIV DNA and cell-associated (CA)-RNA measured as copies per 10^6 cell equivalents.

Time	Day of ART release		Week 2		Month 4.5		Month 7.5 (1 week post CD8 depletion)			Month 8 (2 weeks post CD8 depletion)		
Tissue	PBMC	PBMC	PBMC	PBMC	LN	LN	PBMC	LN	LN	PBMC	LN	LN
Test	DNA	CA-RNA	DNA	CA-RNA	DNA	CA-RNA		DNA	CA-RNA		DNA	CA-RNA
T505	neg	neg	neg	neg	neg	neg	neg	neg	neg	neg	neg	neg
T509	neg	neg	neg	neg	neg	neg	neg	neg	neg	neg	neg	neg
T510	neg	neg	neg	neg	neg	neg	neg	neg	neg	neg	neg	neg
T508	neg	neg	neg	neg	neg	6	neg	neg	neg	neg	neg	neg
