# Supplementary material for: Immunotherapy with DNA vaccine and live attenuated rubella/SIV gag vectors plus early ART can prevent SIVmac251 viral rebound in acutely infected rhesus macaques
Source: PLoS One. 2020 Mar 4;15(3):e0228163. doi: 10.1371/journal.pone.0228163 (PMC7055890; doi:10.1371/journal.pone.0228163)
Supplement: S2 Table — (PDF) [file pone.0228163.s008.pdf]

S2 Table. Viral load in lymph nodes post infection, as measured by ddPCR.

Animal	Group	Avg. copies per 10^6 cell equiv. in LN (week 4)	Avg. copies per 10^6 cell equiv in PBMC (week 7)
T505	vaccine	< LOD (LN)	< LOD (PBMC)
T507	vaccine	< LOD (LN)	< LOD (PBMC)
T509	vaccine	< LOD (LN)	< LOD (PBMC)
T510	vaccine	< LOD (LN)	< LOD (PBMC)
T506	control	< LOD (LN)	< LOD (PBMC)
T508	control	< LOD (LN)	< LOD (PBMC)
T511	control	< LOD (LN)	< LOD (PBMC)
T512	control	< LOD (LN)	< LOD (PBMC)
E026	unrelated negative control	< LOD (PBMC)	
E038	unrelated negative control	< LOD (PBMC)	
T506	positive control after viral rebound	3524 (PBMC)	
T511	positive control after viral rebound	9753 (PBMC)	

Limit of detection for this assay was 4 copies/10^6 cell equivalents
